# Development of a Culturally Specific Leadership Curriculum through Community-Based Participatory Research and Popular Education

**DOI:** 10.31372/20200502.1086

**Published:** 2020

**Authors:** Connie K. Y. Nguyen-Truong, Jacqueline Leung, Kapiolani Micky

**Affiliations:** a Washington State University College of Nursing in Vancouver, Washington, United States; b Micronesian Islander Community and Oregon State University, College of Public Health and Human Sciences in Global Health, United States; c Micronesian Islander Community

**Keywords:** community-based participatory research, popular education, leadership, leaders, Micronesian islanders, community and academic partnerships, capacity building, curriculum

## Abstract

**Background:** The purpose of this innovative capacity building pilot project was to develop, implement, and evaluate a nine-workshop curriculum, *Rekki Lemnak [Thinking of] Parent Leadership*, to prepare community and academic partners for community organizing within the Micronesian Islander community. The purpose of the partnership was to build team leadership and research capacity to lay a foundation for implementing a change in healthcare and school systems. Working collaboratively helped ensure access to shared leadership through the learning by doing approach, enabling a culturally responsive method to build a sustainable partnership.

**Approach:** Community-based participatory research and Popular Education tenets and reflection were used as a guide in the development of the *Rekki Lemnak [Thinking of] Parent Leadership* curriculum. Nine workshops (two hours for eight workshops and three hours for one workshop) were held over a period of a year. Community and academic partners developed the learning objectives, capacity building topics, experiential activities, and an evaluation on the strengths and areas for improvement. The partnership consisted of seven Micronesian Islander parent leaders who are residents from the community at large, the Micronesian Islander Community organization including the Executive Director who is a community primary researcher and certified community health worker, and a Micronesian Islander-certified community health worker staff member, and the academic primary nurse researcher and another academic nurse researcher from Washington State University. A range from five to 10 partners with an average of eight attended the workshops, of which an average of five Micronesian Islander parent leaders attended the workshops. Community partners from the Micronesian Islander Community organization and the academic primary nurse researcher co-led four workshops. Community partners from the Micronesian Islander Community organization and MI parent leaders led two workshops respectively; academic nurse researcher partners led one workshop.

**Outcomes:** We identified three main themes: initially shy and humble MI parent leaders who through their participation transformed to empowered voices, togetherness—coming from different Islands and academia, and the need for more outreach to Micronesian Islanders.

**Conclusions:** Key elements of the *Rekki Lemnak [Thinking of] Parent Leadership* curriculum may be translatable to other community and academic partnerships. Culturally responsive research is more than a process in conducting a study. This requires an ongoing investment to establish and sustain authentic partnerships to conduct research with MI communities.

Community-based participatory research (CBPR) is an evidence-based approach toward bridging the relationship between community partners and academic researchers that addresses a community identified need. This is a process of shared authority between community members and academic researchers as partners throughout the research process: starting from idea generation, data collection, dissemination, and the implementation of research findings (Vaughn, Jacquez, Lindquist-Grantz, Parsons, & Melink, 2017). Historically, CBPR methods primarily take place in marginalized communities due to the time needed to build trust within the communities. CBPR methods include power sharing between academic researchers and community members, enabling academic partners to establish credibility and trust within marginalized communities (Israel et al., 2010). Generally, CBPR methods have been shown to be successful in engaging minority, immigrant, migrant, and refugee populations in research to reduce health disparities (McElfish et al., 2018). In this article, we described the Micronesian Islander (MI) community context; the Micronesian Islander Community organization and Washington State University College of Nursing partnership; and the development, implementation, and evaluation of a nine-workshop series on a culturally specific leadership training curriculum through the use of CBPR and Popular Education tenets for the MI community. The capacity building pilot project took place over the course of one year. Building capacity and trust between MI community leaders and academic researchers as partners required time to develop into a long-term sustainable partnership.

## Community Context

One of the fastest growing communities in the United States (U.S.) includes Native Hawaiians and Pacific Islanders, estimated at close to 1.5 million as of 2016 (U.S. Census Bureau, 2018). Newer immigrants include MI from U.S. associated Pacific Islands (USAPI; Liu, Blaskdell, & Aitaoto, 2008). The six islands have a unique formal relationship with the U.S. government and consist of the following islands: American Samoa, Guam, the Commonwealth of the Northern Mariana Islands, the Federated States of Micronesia (including the four island states, Chuuk, Kosrae, Pohnpei, and Yap), the Republic of Palau, and the Republic of Marshall Islands (Aitaoto, Braun, Estrella, Epeluk, & Tsark, 2012). The MI community has different citizenship statuses within the U.S. In this article, we focused on MIs from the Compact of Free Association (COFA) nations: The Federated States of Micronesia (FSM) and the Republic of Marshall Islands. We did not have MIs from Palau participate in our program. COFA citizens are diverse in language and culture. The FSM alone is composed of more than 600 islands and atolls located across an ocean expanse of 18,000 miles and was settled several thousand years ago by ancient people sailing east from Asia and north from Polynesia (Government of the FSM, n. d.). Eventually, the region became colonized by Spain and Germany. After World War I, the Micronesian nations became a trust territory under Japan. After World War 2, the region from 1947 to 1984 became known as the United Nations Trust Territory of the Pacific Islands to the U.S. (Thomas, 2019). This historical context about the relationship between the Micronesian islands and the U.S. government and the origin of the U.S. presence are important to understand when working with the MI community.

Between 1946 and 1958, the U.S. detonated nuclear weapons on several of the atolls in the Marshall Islands, causing radiation poisoning and the destruction of their respective homelands (Pervis et al., 2016). Individuals who inhabited test sites were relocated while those who lived nearby were not relocated and were exposed to significant radiation exposure in the environment, from what they ate to the air they breathed (McElfish, Hallgren, & Yamada, 2015). An unanticipated impact was the impact the radiation would have on the lands. Marshallese Islanders were forcibly removed from their homes and placed on other islands, with a number who nearly starved to death due to inadequate food crop access (Barker, 2012). The U.S. government examined the effects of the radiation without informing the communities that unfortunately caused significant health issues to the community (McElfish et al., 2015). During the Cold War, the U.S. territory in Micronesia became the prime location to exert U.S. military power (Thomas, 2019). Researchers must understand the impact of the MIs’ historical trauma to build an authentic relationship with the MI community.

Eventually, the COFA nations signed a series of treaties with the U.S. government in 1986 for the Republic of Marshall Islands and the FSM and in 1994 with the Republic of Palau that enabled each island to become independent nations, with certain responsibilities assigned to the U.S. government (McElfish et al., 2015). Under the COFA agreement, COFA citizens were granted broad immigration rights to live, work, attend school, and have access to social and health services in the U.S. with a valid passport and I-94 without the use of a visa. In return, the U.S. has exclusive use of and access to strategic military defense points on the respective island nations for missile testing and space operations until 2066 while providing financial aid to the respective island nations until 2023 (McElfish et al., 2015; Pervis et al., 2016). The treaties were partially established to compensate for the nuclear weapons tests on the Marshall Islands. Radiation exposure and loss of lands, the loss of cultural knowledge and ties, and the rise of ocean water due to global warming have resulted in the inability to grow local food and have made the MI community more reliant on processed foods (Pobutsky, Buenconsejo-Lum, Chow, Palafox, & Maskarinec, 2005; Riklon, Alik, Hixon, & Palafox, 2010). The introduction and adaptation to Western processed foods have led to an exponential increase of lifestyle associated diseases including obesity, diabetes, cancers, and cardiovascular diseases (Pobutsky et al., 2005; Riklon et al., 2010).

Before 1996, COFA citizens residing in the U.S. had access to low-cost medical services through state Medicaid, as they were recognized as legally residing noncitizen nationals (McElfish et al., 2015). However, in 1996, the Personal Responsibility and Work Opportunity Reconciliation Act (PRWORA, welfare reform act) disqualified COFA citizens from most means-tested federal benefits, including Medicaid. COFA citizens were excluded as a category of *qualified immigrants* for eligibility purposes (McElfish et al., 2015). Low-income COFA citizens are not eligible for state Medicaid programs unless U.S. states pass extensive Medicaid programs that provide health care access to COFA citizens, such as the COFA Premium Assistance Programs in both Oregon and Washington. The result of the conversations within the MI COFA communities leads to the development of this leadership curriculum in this article. We later highlighted the struggle of the MI COFA community in understanding the importance of accessing health services such as prenatal care and learning to advocate for their children within the Salem-Keizer public school systems.

## Community and Academic Partnership

The 2010 Census reveals that Pacific Islanders are the fastest growing racial group in Oregon at a 68% increase, with COFA citizens as a significant driver. With an estimated 3,000 COFA in Oregon, Oregon is home to the 5th largest population in the country (Oregon Department of Education, 2014). In the Salem-Keizer School District, translation services are offered for two of the Micronesian languages: Chuukese and Marshallese (Salem-Keizer Public Schools, 2017–2019).

The Micronesian Islander Community organization, a MI specific community organization, was established in 2011 and operates as a 501c3 tax exempt organization. The Micronesian Islander Community organization is a trusted source known for providing culturally specific social and community health services and related trainings. In 2018, MI parents, who are residents in the MI community at large, reached out to the Micronesian Islander Community organization expressing interest in creating trainings focused on health and education. MI parent leaders did not have prior experience with research. In that same year, the Micronesian Islander Community organization connected with the academic nurse research partner, Washington State University College of Nursing, who has specialties in nursing, leadership development, CBPR, Popular Education, and marginalized communities. The Micronesian Islander Community organization, academic nurse research partner, and MI parent leaders in the Pacific Northwest region of the U.S. built a trusted relationship over the course of a year. Through our community and academic partnership, our team recognized the impact of the MIs historical trauma and their distrust of outsiders, and therefore, the importance of building an authentic relationship with the MI community. Through previous conversations, we learned that the MI community is shy and humble (^1^Nguyen-Truong, ^1^Leung, Micky, & Nevers, in press). The MI community expressed interest in leadership workshops. The aim of the pilot project was to build leadership skills within the MI community as partners through the use of CBPR and Popular Education tenets. The project answered the following question: What are concerns among MI parent leaders related to health and education? The result of our collaborative work was the development, implementation, and evaluation of a nine-month culturally specific leadership curriculum as a pilot project created in partnership with academic and community partners.

The academic and Micronesian Islander Community organizations worked together with MI parent leaders to develop the curriculum, entitled, *Rekki Lemnak [Thinking of] Parent Leadership*. This curriculum was designed for building leadership and research capacity through community engagement. The process of designing and implementing the curriculum enabled the MI community and academic partners to build team leadership and research capacity to understand how MI parents navigated access to prenatal healthcare and how families navigated through the U.S. public school system. We worked collaboratively to ensure access to shared leadership through learning by doing, building community through identifying existing resources, and creating the foundation for building a sustainable culturally responsive partnership. The Washington State University has determined that the project (IRB # 17203-001) satisfies the criteria for Exempt Research at 45 CFR 46.101(b)(2).

## Approach

### Design

**Pedagogy.** Community-based participatory research emerged in recent decades as a transformative research paradigm that bridges the gap between science and practice through community engagement and social action to increase health equity (Yang et al., 2019). We used a central tenet of CBPR: building a partnership between community members and academic researchers dependent on the team’s ability to lead and work together to build trust and capacity to respond to a community identified need. We focused on sustainability in building and nurturing a partnership, regarding that the partnership is a long-term dynamic process that builds upon the foundations of trust, colearning, and commitment (Israel, Eng, Schulz, & Parker, 2012). The CBPR perspective includes community engagement starting with outreach to redress the distrust and the inherent power imbalances of the research enterprise (Wallerstein, Duran, Oetzel, & Minkler, 2018). Community and academic partners decided and developed the learning objectives, capacity building topics, experiential activities, and the evaluation of the *Rekki Lemnak [Thinking of] Parent Leadership* curriculum to facilitate learning through building team leadership and research capacity.

We used the following Popular Education tenets because it focuses on empowerment and consideration of what partners bring to the table (^1^Nguyen-Truong, ^1^Fritz et al., 2018; Nguyen-Truong, Tang, & Hsiao, 2017; Wiggins, 2012). We learned with our heads (i.e., cognitive), hearts (i.e., affective), and bodies (i.e., psychomotor). Together, the community and academic partners created a learning environment that enabled partners to be open, develop trust, and to share their perspectives and personal narratives with one another. We promoted equality between the partners such as validating knowledge gained through life experience. Life experiences are equally important as knowledge gained through formal education. Community engagement through active learning rather than passive learning and the use of artistic teaching modalities occurred throughout the curriculum. Learning progressed in a spiral manner (Whelan, 2005). The community started with experience and knowledge in what partners already knew, and from there, identified patterns in experience and learning, while adding new information, practicing new skills learned, and applied new skills to building team leadership and research capacity. Individual and group reflection occurred through learning together.

### Implementation

**Setting and partners as co-learners.** The trained MI certified Community Health Worker (CHW) staff, who identifies as Chuukese and speaks English and Chuukese, conducted outreach and recruited 11 MI parent leaders as participants who are known by the community partners from Micronesian Islander Community organization network from an urban area of the U.S. Pacific Northwest region. Of the initial 11 MI parent leaders who started the work sessions, three decided not to participate due to a work schedule conflict. The inclusion criteria for MI parent leaders included: participants who self-identified as a MI parent or caregiver with a child or had children less than eight years old including pregnant MI, who spoke and understood English or a MI language. Details of the sociodemographic characteristics of the MI parent leaders are described elsewhere (^1^Nguyen-Truong, ^1^Leung et al., 2020). The MI parent leaders chose to communicate in English and most reported speaking English well (6) including one reported very well (1), and self-identified as females. Seven MI parent leaders (five Chuukese and two Marshallese) were self-identified as mothers with a child or had children less than eight years old, including one who was pregnant, and were residents from the community at large. The Executive Director is a community primary researcher and certified CHW, and speaks English, and the MI CHW staff are from the Micronesian Islander Community organization. The academic nurse researchers, of which one is a primary researcher and is Vietnamese and speaks English and Vietnamese and another is White non-Latin and speaks English, are from Washington State University.

Nine workshops, of which eight workshops were two hours in length and one workshop was three hours in length, were held over a period of a year to build team leadership and research capacity between partners. The workshops were held in a rented community conference space in an urban area of the U.S. Pacific Northwest region. Between five and ten partners with an average of eight attended the workshops, of which an average of five MI parent leaders attended the workshops. Of the partners, seven included MI parent leaders and the Executive Director/a primary researcher/CHW from the Micronesian Islander Community organization were unable to attend one or more workshops due to extenuating medical, family, or community obligation reasons and met with the MI CHW staff from the Micronesian Islander Community organization for a review. Two MI parent leaders withdrew from participation after the first workshop due to extenuating family reasons. Community partners from the Micronesian Islander Community organization and the academic primary nurse researcher co-led four workshops. Community partners from the Micronesian Islander Community organization and MI parent leaders led two workshops respectively; academic nurse researcher partners led one workshop.

See Table 1 where we described key elements of the implementation of the *Rekki Lemnak [Thinking of] Parent Leadership* curriculum. This includes the (a) learning objectives, (b) capacity building topics, (c) experiential activities, d) mean evaluation scores, (e) community and academic partners, and (d) resources and references that informed the experiential activities.

**Table 1:** Rekki Lemnak [Thinking of] Parent Leadership Curriculum: Learning Objectives, Capacity Building Topics, Experiential Activities, Mean Evaluation Scores, and Community and Academic Partners

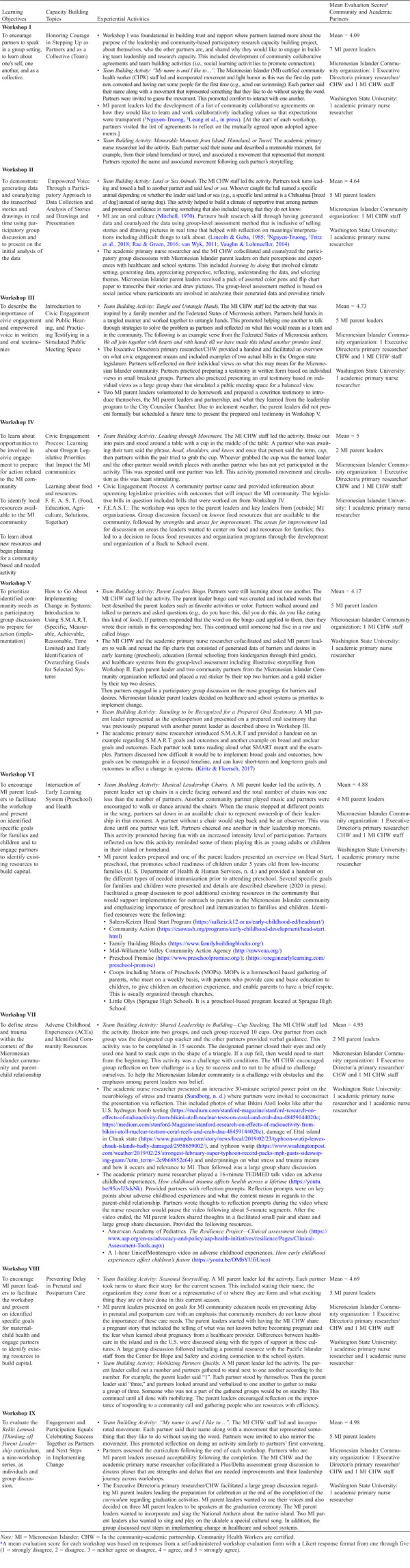

### Evaluation

To properly evaluate the outcomes of the *Rekki Lemnak [Thinking of] Parent Leadership* curriculum, we used a three-step data collection and analysis. We asked community and academic partners to assess the *Rekki Lemnak [Thinking of] Parent Leadership* curriculum at the end of each workshop with the self-administered workshop evaluation form that used a Likert response format. We specifically asked partners who are MI parent leaders to assess acceptability following the completion of the curriculum with the self-administered acceptability of a program scale that also used a Likert response format. Further, we used the plus/delta assessment and discussed as a group, the shared leadership journey as partners in terms of the pluses that are strengths and deltas that are needed improvements in the pilot implementation of the curriculum. We recognize limitations in using Likert response formats with low literacy communities in other researchers’ previous studies. Specifically, the use of Likert response formats among immigrant communities is controversial because there are extraneous variables that may impact responses. This includes education level, acculturation, and the country of origin (D’Alonzo, 2012). There remains little agreement about the most appropriate way to measure the outcome (D’Alonzo, 2012). However, the use of adapted instruments that has been used in other immigrant communities for individual assessment and a group-level assessment enabled us to provide a deeper, enriching context of the materials that were provided in the curriculum.

A primary academic nurse researcher went over the instructions for the self-administered workshop evaluation form and self-administered acceptability of a program scale. This included the purpose, anonymity, and confidentiality, having read through the items and response options and what it meant, and having an opportunity to ask and answer questions. The MI CHW staff emphasized the importance of being honest in responses. No one expressed concerns about not being able to read or understand the items. The primary academic nurse researcher and the MI CHW staff were available to go through the items such as the meaning of the response statements.

We adapted a self-administered workshop evaluation form that consists of eight items with a Likert response format from one to five (1 = strongly disagree, 2 = disagree, 3 = neither agree or disagree, 4 = agree, and 5 = strongly agree; Nguyen-Truong, Tang et al., 2017). In this self-administered workshop evaluation form, we also provided an open-ended option to provide written comments/opinions. There were no written comments/opinions from the form to analyze. The following are the ranges of mean scores of the evaluations: (1) *The workshop objectives were clear to me* (mean = 4.2 to 5), (2) *The workshop activities stimulated my learning* (mean = 3.88 to 5), (3) *The contents of the workshop were meaningful* (mean = 4.13 to 5), (4) *The difficulty level of this workshop was appropriate* (mean = 4.13 to 5), (5) *The pace of this workshop was appropriate* (mean = 3.88 to 4.86), (6) *The facilitators/speakers were well prepared* (mean = 4.2 to 5), (7) *The facilitators/speakers were helpful* (mean = 4.2 to 5), and (8) *I will be able to use what I learned in this workshop* (mean = 4.07 to 5). See Table 1 for a mean evaluation score for each workshop.

We adapted a self-administered 10-item accept-ability of a program scale (Nguyen-Truong, Tang et al., 2017) that contains process questions related to cultural appropriateness of the content and response to the curriculum. Item responses are from one to four (1 = strongly disagree, 2 = disagree, 3 = agree, and 4 = strongly agree). Item examples include: *The leadership program increased my knowledge of community building, I enjoyed attending this leadership program, The length of this leadership program was acceptable*, and *I think this leadership program will be well received by other people of my age and from my homeland*. The project is considered acceptable if at least 80% of participants assessed the project positively (Nguyen-Truong, Pedhiwala et al., 2017; Wahab, Menon, & Szalacha, 2008). The project was assessed positively by 100% of MI parent leaders.

The MI CHW staff and the academic primary nurse researcher coconducted the Plus/Delta assessment that was adapted from O’Connell and Vandas (2015). We used an open-ended, semistructured guide and collected field note based data on the group discussion. For example, “What are the strengths in this leadership curriculum that help to prepare you for leading a change in a healthcare and education systems?” (pluses) and “Describe your recommendations for what could be improved in the leadership curriculum for participation by Micronesian Islander parents/families” (deltas). In addition, we also asked MI parent leaders to “Describe a story of your journey in this leadership curriculum and your growth with other parent leaders and the partners from the Micronesian Islander Community organization and nurse researchers.” We debriefed, verified, and interpreted the field note based data with partners to ensure trustworthiness (Lincoln & Guba, 1985). Partners included: five MI parent leaders, the Executive Director/a primary researcher/CHW and the MI CHW staff from the Micronesian Islander Community organization, and the academic primary nurse researcher.

## Discussion on Outcomes

We identified three main themes from the pilot implementation of the curriculum: from initially shy and humble MI parent leaders who through their participation transformed to empowered voices, togetherness—coming from different Islands and academia, and the need for more outreach to Micronesian Islanders.

### Initially Shy and Humble and Through Participation Transformed to Empowered Voices

We learned with a deeper understanding from MI parent leaders and community partners from the Micronesian Islander Community organization that MI are shy and humble due to their cultural upbringing. MI women are generally reserved and quiet. In traditional MI society, leaders are chiefs, who are often male, and carried through matrilineal lines (Poyer, Carucci, & Falgout, 2016). However, the initial contact by the Spanish destroyed indigenous leadership in various parts of the MI islands (Poyer et al., 2016). Germany implemented direct rule and altered land tenure. Later, Japan after World War I established a “colonial service that used chiefs for some administrative tasks but also pushed acculturation and an educational meritocracy, along with economic development and large-scale immigration” (Poyer et al., 2016, p. 109). Chiefs among Japanese-ruled Micronesia varied greatly in authority (Poyer et al., 2016). Today, its leadership structure is fractured, as more MI leave the islands for work, health, or family reasons, for Guam, Hawaii, or the U.S. mainland.

At the start of the implementation of our workshop, we observed with MI parent leaders who often lowered their heads and covered their faces with a paper or with their hand or arm prior to speaking or while speaking when they were invited to speak and when they spoke. As said by a MI parent leader, *“We are shy. I am trying.”* The MI CHW staff said, *“The parent leaders are very shy and did not have the courage to stand in front of many or few people. They are very humble even though they know what to do but they are very respectful and humble.”* Overtime, we observed that MI parent leaders lowered and covered their faces less frequently after five workshops when we began to focus on leadership topics that built upon empowering voices as individuals and as a collective. Community and academic partners emphasized that the interactive team building activities encouraged people to feel safe to interact comfortably with one another and to connect while having enjoyment while learning collaboratively. This adds to our previous capacity building work where we found community partners from Asian communities including Chinese, Filipino, Japanese, Korean, Taiwanese, and Vietnamese had felt safe and joy to engage in experiential activities through interactive social learning activities that centered on team building (^1^Nguyen-Truong, ^1^Fritz et al., 2018; ^1^Nguyen-Truong, ^1^Leung et al., in press; Nguyen-Truong, Tang et al., 2017). MI parent leaders shared what they learned about themselves and reflected on their leadership journey as they began to lead experiential activities. Furthermore, MI parent leaders expressed feeling their confidence increase. A MI parent leader said, *“Discovering who I am, where I’m from through storytelling.”* Another parent leader said, *“Grew confidence by speak up, stand-up, voice out.”* Previous researchers found in their work among Pacific Islanders that investment in leadership development led to effective leaders and champions that ensured adoption of interventions (David et al., 2013). Community and academic partners found overall that the length of time for the workshops were necessary and protective for leadership and research capacity building. This helped MI parent leaders to be engaged as they experienced a cultural shift to feeling confidence in speaking.

### Togetherness: Coming from Different Islands and Academia

Community partners identified language barriers, having a limited understanding of available resources in the community, and the cultural acceptable practice of observing silence among MI community members, and not interacting with other MI communities other than their own to be among the top barriers regarding early learning and healthcare use and understanding. The MI CHW staff said, *“English is not our native language…don’t be shy to speak English. We are here to learn to survive.”* Community and academic partners expressed having accepted one another’s differences, including one’s culture even when they are from different islands, and in working with an academic institution. Building trust was necessary to be able to discuss issues and potential solutions. The findings are similar to previous researchers in their work with Pacific Islanders regarding the crucial involvement of community partners to build trust and investing in long-term relationships to humanize the connection (Chung-Do et al., 2016; David et al., 2013; Kwan et al., 2017).

Working together as community and academic partners helps to identify locally available existing resources and to discuss how to leverage resources (Wallerstein et al., 2018). However, culturally specific considerations are a must. For instance, in our current capacity building pilot project, community and academic partners discussed challenges the MI community faced to access fundamental resources from organizations, the government, and from the school system. MI parent leaders felt embarrassed to seek existing resources. *“They have so much pride that lead to not wanting to go to other places that they know they will see other community members. They feel insecure that people will gossip about them or make fun of them or even think that they don’t have money or they are poor.”* MI parent leaders said that solutions need to address both the individual level combined with outreach in the MI community. *“…[need to] put down the pride you [referring to MI community members] have and start thinking about your family…If we have a day to have outreach within the community and spend time educating them or giving them a list of resource they need including phone numbers.”* Although partners can create an inventory of existing knowledge of resources available to the MI community, it is important to consider culturally specific concerns that community members have about the use of resources. MI parent leaders also talked about wanting community members to know who they are and that they are here to help through authentic messaging and providing their contact information. Language barriers are a struggle within the MI community. Community members may not seek resources even if they knew of said resources if they do not feel confident about their ability to speak English. Community partners expressed the need to provide translated materials in their respective languages.

### The Need for More Outreach to Micronesian Islanders

Partners need to identify and discuss mutual benefits (Wallerstein et al., 2018). Community and academic partners discussed the need for more MI community involvement in community organizing efforts to build capacity for leadership and research. This included encouraging participation from other MIs by asking and responding to community members’ needs. For example, partners discussed needing to know about community resources to effectively support the community. MI parent leaders expressed appreciation for knowing what the existing community resources are so that this can inform teamwork for leading a system change in early learning and healthcare and to share the knowledge with other MIs. *“Sharing what I learned with my community.”* *“Gain knowledge…outside groups about community”* [e.g., Family Building Blocks, an early learning system].

While discussing the curriculum, MI parent leaders expressed wanting more than one workshop each month. Most preferred to attend two workshops a month for at least two hours per workshop session. To develop two 2-hour workshops each month would require significant time and flexibility on behalf of all partners to ensure a twice monthly workshop could take place. Each workshop was scheduled based on the availability of Micronesian Islander Community-academic partners and MI parent leaders as partners. There were several date changes due to illnesses and inclement weather (e.g., snow). These factors must be factored in when developing a curriculum.

We noted that workshops I and V had above average scores. An above average score on the first workshop is acceptable, given that it was the initial meeting and the start of trust building. As most participants did not know one another, it was a starting meeting for most participants. The workshop included terminology and activities that were new to the participants. It was also a base building opportunity for the community. Workshop V had the second above average score. This is likely because the topic was building knowledge on what the MI parent leaders were interested in and an introduction to S.M.A.R.T. goals and outcomes, a brand-new term the community was not familiar with (i.e., specific, measurable, attainable, realistic, time limited). It may be beneficial to introduce S.M.A.R.T. goals and outcomes earlier in the workshop series. We built on this workshop as we continued to work as partners with a broader context on the purpose of learning/understanding S.M.A.R.T. goals and outcomes.

### Future Recommendations

A limitation of the *Rekki Lemnak [Thinking of] Parent Leadership* curriculum is the need to schedule workshops that accommodated partners’ schedule needs. We learned the importance of being flexible with meeting times, as parent leaders preferred to meet in the evenings after 5:00 pm Pacific time. Timing needs to be decided within partnerships to allow for maximum team building, participation, group reflection, and discussion. The Executive Director (a primary researcher), CHW, MI CHW staff, academic primary nurse researcher, academic nurse researcher, and MI parent leaders who led and participated in the workshops nearly all had a formal education background and all self-identified as females and spoke and understood English. In addition, MI parent leaders were mostly middle aged and lived in the U. S. between two years to about two decades. Cultural expectations in building trust and capacity may be different depending on age, education attainment, immigration status, and gender for other MI and racial-ethnic groups. This pilot project was implemented in an urban area in the U.S. Pacific Northwest region. We recommend community and academic partnerships discuss leadership and research capacity building needs as the experience can differ for other partnerships and for communities in other geographic locations. We recommend translating materials and evaluation tools in MI languages for future use.

## Conclusion

We described the development, pilot implementation, and evaluation of an innovative leadership and research capacity building pilot project. The development of the culturally responsive *Rekki Lemnak [Thinking of] Parent Leadership* curriculum for leadership development and research capacity was necessary because there is no leadership development programs specific for MI parent leaders’. Our curriculum helped MI parent leaders develop confidence in speaking about community needs as this is not common practice in the MI culture. Key elements of the *Rekki Lemnak [Thinking of] Parent Leadership* curriculum may be translatable to other community and academic partnerships. We identified three main themes regarding building leadership and research capacity from the pilot implementation of the curriculum: shyness and humbleness to empowered voices, togetherness—coming from different islands and academia, and a need for more outreach to MI. Building capacity between MI community partners and academic researchers as partners took one year to build long-term sustainability. Being culturally responsive requires time to build leadership and research capacity holistically. Culturally responsive research is more than a process in conducting a project. This requires an ongoing investment to establish and sustain authentic partnerships to conduct research with MI communities.

## Acknowledgments

The authors would like to thank Jennifer Nevers, BSN, RN, a PhD Student, at Washington State University College of Nursing in Spokane, for collaboration and subject expertise in the neurobiology of stress and trauma. The authors are appreciative of the anonymous peer reviewers for assistance.

## Declaration of Conflicting Interests

The authors declared no potential conflicts of interest concerning the research, authorship, or publication of this article.

## Funding

Dr. Jacqueline Leung, JD, MS, CHW, Dr. Connie Kim Yen Nguyen-Truong, PhD, RN, (Alumnus PCCN), and Kapiolani Micky, BA, CHW received the Health and Education Fund Impact Partnerships #18-02376: Northwest Health Foundation, Meyer Memorial Trust, Care Oregon, Kaiser Permanente Northwest, and Oregon Community Foundation.
